# Scientists’ Prioritization of Communication Objectives for Public Engagement

**DOI:** 10.1371/journal.pone.0148867

**Published:** 2016-02-25

**Authors:** Anthony Dudo, John C. Besley

**Affiliations:** 1 Stan Richards School of Advertising & Public Relations, Moody College of Communication, The University of Texas at Austin, Austin, Texas, United States of America; 2 Dept. of Advertising & Public Relations, Michigan State University, East Lansing, Michigan, United States of America; Queen Mary University of London, UNITED KINGDOM

## Abstract

Amid calls from scientific leaders for their colleagues to become more effective public communicators, this study examines the objectives that scientists’ report drive their public engagement behaviors. We explore how scientists evaluate five specific communication objectives, which include informing the public about science, exciting the public about science, strengthening the public’s trust in science, tailoring messages about science, and defending science from misinformation. We use insights from extant research, the theory of planned behavior, and procedural justice theory to identify likely predictors of scientists' views about these communication objectives. Results show that scientists most prioritize communication designed to defend science from misinformation and educate the public about science, and least prioritize communication that seeks to build trust and establish resonance with the public. Regression analyses reveal factors associated with scientists who prioritize each of the five specific communication objectives. Our findings highlight the need for communication trainers to help scientists select specific communication objectives for particular contexts and audiences.

## Introduction

Recent years have witnessed an upsurge of attention to scientists as public communicators. Much of this attention stems from leaders from within the scientific community who encourage scientists to boost their communication efforts to help build rapport with the public and ensure that their views contribute to policy-making (e.g., [1–5]). Simultaneously, research on scientists as public communicators is becoming more common. This research is providing a clearer sense of how often scientists engage with lay audiences and the characteristics that drive these communication efforts (e.g.,[6–9]). Addressing these baseline questions has advanced both intellectual activity and best practices relative to scientists’ public engagement. But this work also has highlighted new questions that necessitate increasingly granular research. Given the state of literature, it seems an appropriate time to help supplement the field’s understanding of the descriptive aspects of engagement (e.g., quantity, modality, etc.) with attempts to understand what scientists hope to affect through their public communication efforts.

In this study we address this issue by focusing on a handful of potential communication objectives that scientists may pursue through engagement as a mechanism for having an impact on citizen attitudes and behavior. We believe that exploring scientists’ communication objectives—which are operationalized below—represents an important area of research because strategic objective-setting can influence the effectiveness of engagement efforts. Specifically, we assess how one population of scientists views five discrete objectives for online public communication and identify key predictors associated with assessments of each of these objectives. Our analyses are based on a sample of members from the world’s largest general scientific society (*N* = 390) and are guided by extant public engagement research, the theory of planned behavior [10] and procedural justice theory [11],

## Literature Review

### Scientists’ public engagement

Leaders in the scientific community are calling on American scientists to meaningfully engage with their fellow citizens. The hope is that such interactions can enhance the science-society relationship by bringing “scientists into closer proximity with their fellow citizens … [to] give each group a far better understanding and greater empathy for the perspective of the other” [3]. Such calls have a long history [12,13], and social scientific research on this topic—commonly referred to as public engagement of science (PES)—has escalated somewhat rapidly.

One fertile area of PES scholarship has focused on identifying factors associated with scientists’ participation in outreach, similar to how the political science literature has looked at what gets citizens to engage in politics [14]. This research has unearthed a number of factors associated with scientist engagement, among them communication self-efficacy and autonomy [7, 8, 15–17],attitudes toward engagement [3,7,15,18,19]; scientific status [3, 6–8,18, 20], presumed media influence [15,16], and norms [17].

Overall, these studies have focused on scientists’ views about engagement, willingness to engage, or past engagement. They have not addressed what scientists mean when they say, for example, that they are willing to interact with the public. One strong possibility is that scientists who say they were willing to engage really mean that they are willing to perform one-way outreach designed to transfer factual information. Inasmuch as such education is but one potential communication objective, there is an opportunity to build on the extant research by exploring what objectives underlie scientists’ engagement efforts.

### Scientists’ communication objectives: Moving beyond the traditional focus on information sharing

The belief that increased knowledge will lead to support for science—what is often called the “Deficit Model” [21–22]—has historically been widespread within the scientific community [23]. Knowledge, according to this view, leads to a range of attitudes and behavior that contribute to the health of the scientific enterprise. Deficit model thinking, however, has faced widespread criticism as the evidence suggests that knowledge is rarely a dominant driver of attitudes toward issues involving science, whether it be climate change, emerging technologies, or vaccinations. The evidence instead points to the key role that affective factors play in shaping views about science. Such factors include the degree to which individuals see decision-makers as sharing their values (e.g., [24]) and being trustworthy or fair (e.g., [25]), as well as their overall views about science in society (e.g.,[26]). Communication scholars now emphasize that positive beliefs about science and scientists are more likely to stem from high quality interactions with likable and engaging scientists who are willing to listen [21, 27].

For our purposes, the limits of imparting knowledge as a communication objective means that a scientist may want to devote more resources to achieving other objectives. This does not mean that seeking to increase knowledge is unimportant; it only suggests that a strategic approach to communication would involve putting adequate emphasis on, for example, demonstrating that one is listening to others, choosing language that resonates with what individuals are already thinking about, or highlighting common-ground between scientists and non-scientists. These objectives, for example, are more consistent with bi-directional communication, which is a mode of communication that research fields including political science (e.g.,[28]) and public relations (e.g.,[29]) have shown to lead to better engagement outcomes.

It is also important to recognize that in focusing on communication objectives, we are not (yet) focusing on what might be called scientists’ ultimate goals. These might include societal-level goals such as marshaling support for specific policy positions or strengthening the scientific community through youth recruitment, and individual-level goals such as enhancing or adjusting one’s career trajectory. The expectation is that it may be possible to eventually connect communication objectives to these ultimate goals, but our analysis in this paper represents a necessary first step.

### The Current Study

Our study is motivated by two opportunities. The primary opportunity is that, although the science communication field is coming to understand the factors associated with scientists’ public engagement, we have not asked how such factors shape the explicit communication objectives that scientists hope to achieve when they interact with their fellow citizens. Specifically, there is an opportunity to understand what might lead scientists to approach communication as a chance to strategically engage with others to achieve specific outcomes versus engaging non-strategically in the hope of, for example, simply fixing perceived knowledge deficits. Given that many scientists see the public as misguided and irrational [30–31] there may also be a danger that an over-emphasis on getting specific facts across may lead some scientists to inadvertently interact in ways that fail to communicate respect, or miss chances to build trust and excitement.

A second opportunity is that engagement scholars have developed substantial insight into what it takes to have an impact on how the public views science, but these insights are only beginning to have an impact on the engagement practice [32]. Related fields such as public health—which may include some elements of science communication as part of efforts to foster behavior change—in contrast, are committed to designing interventions based on theory and research that emphasize building knowledge as well as shaping attitudes, norms, and efficacy [33]. A key impetus for this study, therefore, is the opportunity to try to better understand what scientists think about public science communication and then use this knowledge to (re)design training programs so that can overcome the gap between science communication research and practice. With this context in mind, we seek to extend the literature that examines scientists’ as public communicators so as to provide a better sense of what scientists hope to achieve when they communicate. Specifically, we aim to see how one important scientist sample prioritizes five different communication objectives and then test for associations with potential predictors of these objectives. Views about these five specific types of communication objectives were chosen as the criterion variables based on scholarship addressing the types of impacts that can be expected from public engagement. As noted, these include the degree to which scientists see value in online public communication aimed at (1) informing (i.e., educating) others about science (2) exciting others about science, (3) ensuring others see scientists as trustworthy, (4) framing or shaping messages to resonate with people’s existing views, and (5) defending science from perceived misinformation. The first of these objectives references the traditional emphasis on public communication designed to inform as discussed above. The others, however, represent communication objectives that require more explanation.

Even more straightforward than the objective of providing information, is the communication objective of piquing interest in science. Educating and building interest are traditionally considered two sides of the same coin when it comes to public engagement with science, however these objectives are identified as distinct “strands” of science learning within the influential *Learning Science in Informal Environments* report [34]. The report specifically discusses the unique role that sparking interest and excitement can play in spurring public motivations to seek future opportunities to learn about and engage with science. We therefore included building excitement as a specific communication objective.

We included building trust as a distinct communication objective because it is an expected outcome of quality engagement [35–36]. For example, community relations activities as discussed within strategic communication scholarship are designed to build stakeholder trust [37]. Similarly, research on procedural justice highlights the need to communicate in ways that convey caring and that give the individual a meaningful voice [38]. This suggests scientists who want to build trust should seek to demonstrate to their audiences that they are good listeners and that they care about their communities. Past work has demonstrated the importance of justice perceptions within the context of science (e.g., [39–40]), but have not assessed to what extent scientists consider building trust—operationalized here as procedural fairness perceptions—as an important engagement objective.

Tailored messaging, a fundamental pillar of strategic communication [41], has also been widely discussed within the science communication community in recent years. Based on a surfeit of research showing that individual-level predispositions and heuristics influence perception formation about scientific issues (e.g., [42–43]), scholars have stressed that scientist communicators should consider ensuring that messages resonate with their audiences [44]. The idea has generated backlash from segments of the scientific community, suggesting—anecdotally, at least—that some scientists are hesitant to prioritize tailored messaging. This reticence appears because of concerns that careful crafting of messages may lead to perceptions of scientists engaging in ‘spin’ [45]. Nevertheless, it seems important to assess scientists’ view of this particular objective because of the topic’s prominence in the literature.

Defending science is the final communication objective we sought to examine. Scientists have a long history of criticizing media and have been particularly critical of what they perceive to be rampant inaccuracies within journalists’ coverage of science [46–48]. And there are signs that proponents of science commonly engage in aggressive public communication aimed at individuals who reject the science of climate change, evolution, and vaccines (e.g., [49–50]). Together, this desire to correct inaccuracies and counter anti-science views, may spur scientists to engage in public communication designed to defend science from what they perceive to be public misinformation or attacks. This objective is particularly noteworthy given the research literature showing how aggressive and uncivil discourse can have a variety of negative impacts on respondents’ perceptions of decision-makers and overall decision-making processes [51–53]. This is an especially important objective to study further relative to contentious scientific issues.

Overall, we aim to identify why some scientists may prioritize certain objectives for public communication more than others. We focus on these specific types of public engagement objectives because these objectives represent concrete points of emphasis for scientists who interact with the public. Our first research aim is to see how scientists prioritize these different communication objectives, while taking into account their perceptions of how their colleagues prioritize these objectives. To our knowledge no research has assessed this topic, so we offer the following research questions:

**RQ1:** What objectives do scientists prioritize when communicating with the public?
**RQ2:** To what extent do scientists think their colleagues share these same objectives?

Our second research aim is to identify key factors that are associated with scientists’ valuing of each of these specific objectives. We use the theory of planned behavior (TPB) [10] as a conceptual model to help in the selection of endogenous variables. The TPB has been used extensively in research focused on understanding behaviors related to science, health, the environment, and risk (e.g., [7,15,17,54]). Overall, this research has found empirical support for the TPB within the science communication context, specifically that self-efficacy, attitude, and norms relative to public communication are often associated with scientists’ willingness to engage and/or their actual engagement behavior. It therefore seems reasonable to expect that such variables might also be useful for predicting scientists’ prioritization of specific communication objectives.

We also draw guidance from research on deliberative democracy and the focus it has placed on designing engagement mechanisms that citizens see as legitimate [33,55]. As discussed above, work in this area has pointed to procedural justice theory and its emphasis promoting distributive fairness (i.e., do individuals get what they see as a fair outcome) and procedural fairness (i.e., do individuals feel like they have a voice) [56] in engagement efforts. Specifically, we measure these concepts to see if scientists’ prioritizations of communication objectives are associated with the degree to which scientists see the public with whom the scientist may engage as likely to help or hurt their career (distributive fairness/unfairness) and as likely to treat them with respect and an open-mind (procedural fairness/unfairness).

The perceived ethicality of specific training objectives also seems likely to influence how scientists prioritize those objectives. As noted, many scientists are wary of communicating in ways that might suggest that personal preferences might bias their research towards their preferred outcomes. Communication scholars, however, have advocated the need for scientists to think strategically about how to best communicate with the public (e.g., [57]). Given this challenge, we included questions addressing the degree to which respondents’ felt that the specific communication objectives were ethical.

Although many of the concepts mentioned above derive from well-established theories and have been tested within science communication research, these concepts have not yet been tested relative to predicting specific communication objectives. The second goal of our study is therefore exploratory, so we propose the following research questions:

**RQ3:** What factors are associated with scientists’ objectives for communicating with the public?

Our focus on online engagement derives from a general interest in this modality as an increasingly important channel for interactions between scientists and the public (Brossard, 2013; Brossard & Scheufele, 2013; Peters, Dunwoody, Allgaier, Lo, & Brossard, 2014) and associated interest by the American Association for the Advancement of Science (AAAS), a key research partner. We felt it was important to ground this initial study in a relatively specific form of engagement, but the discussion below also considers how our findings comport with what is currently known about scientists’ “offline” public engagement.

Similarly, our working understanding of the ‘public’ for the current study is driven by a belief that contemporary science communication should be understood in terms of current debates about the practical and normative value of ‘public engagement’ in democratic societies. This means that the current study conceptualizes the public in a general sense as those who might be involved in non-private “talking, discussing, debating, and/or deliberating” ([58], p. 318) about issues of public importance (e.g., science, in general, or specific science topics). This might include, for example, anyone that a scientist might interact with through programs at a science museum, science festival, or public talk, as well as someone who might encounter that scientist by reading or watching them in news media or through online channels (e.g., websites, Facebook, Twitter, YouTube, etc.). While, it may be useful to investigate the degree to which specific contexts (e.g. topics or situations) might affect scientists’ views about communication, it also seems important to initially understand scientists’ general views about communication objectives. This argument is similar to the argument about the value of considering general attitudes towards science as well as specific attitudes towards scientific topics [59]. Data from the current study could also be used as benchmarks for the study of communication views in the context of specific topics (e.g., objectives when planning communication about climate changes) or approaches (e.g., objectives when planning for a science festival or a public talk).

## Materials and Methods

### Sampling procedure

The data used in this study were collected in an online survey conducted during October and November of 2013. The survey was administered to a sample of 5,000 members of the American Association for the Advancement of Science (AAAS). These AAAS members were chosen randomly, but all met three criteria: Each member (1) was based in the United States, (2) had a Ph.D., and (3) was working for a university. For confidentiality reasons, the AAAS emailed anonymous survey invitations to the sample instead of using traditional mailers. Adhering to the Tailored Design Method [60], three contacts were made with the sample over approximately four weeks with the reminders sent once responses to the previous invitation had slowed. To help strengthen the rate of responses, we offered half of the respondents a 1/200 chance to win a $500 Amazon.com e-gift card while the second half were told we would donate $500 to the AAAS for every 200 responses. The study protocol was approved by the Institutional Review Boards of the University of Texas at Austin (study#: 2013-08-0061) and Michigan State University (study#: x13-854e/APP#i044402).

A total of 390 usable surveys were returned for a response rate of 8% and the incentive options did not appear to have any impact (analysis not shown). This response rate is consistent with other online surveys of expert communities (e.g.,[61]), and in this case, is likely artificially suppressed because the priority for preserving confidentiality meant that it is not possible to adjust the response rate to account for faulty email addresses (i.e. hard- and soft-bounce backs). Given the AAAS’s eligible membership population size of more than 50,000, our sample provides a margin of sampling error of about +/- 5% at the 95% confidence level for the entire sample. Mean comparisons between early and late responders yielded no meaningful distinctions (analysis not shown).

### Measures

Our analyses included eight control variables. The demographics were gender (62% male), age (*M* = 55.95, *SD* = 14.36), and political ideology measured on a 5-point scale from very conservative (1) to very liberal (5) (*M* = 3.97 *SD* = .91). We also controlled for respondents’ professional status via measures of their research productivity and their career level. To measure scientists’ research productivity, respondents were asked to indicate their number of career publications (a six-point scale from 1 [<10 publications] to 6 [>100 publications]) (*M* = 3.52, *SD* = 1.89). Career level was measured by asking scientists whether they consider themselves student- (1), junior- (2), mid-career- (3), or senior- (4) level researchers (*M* = 3.41, *SD* = .80).

We also controlled for respondents’ use of traditional and online media for obtaining news about science. Scientists’ consumption of science news from traditional media sources was measured by asking them to indicate how frequently (on a five-point scale from 1 [never] to 5 [nearly everyday]) they sought news about science via television (including online) (*M* = 2.67, *SD* = 1.30), magazines (including online) (*M =* 3.61, *SD* = 1.22), newspapers (including online) (*M* = 3.76, *SD* = 1.27), and radio (including online) (*M =* 3.31, *SD* = 1.41). A composite measure of traditional consumption of science news was constructed (Cronbach’s α = .66, *M* = 3.32, *SD* = .91). Scientists’ consumption of science news from online-only media sources was measured by asking them to indicate how frequently (on a five-point scale from 1 [never] to 5 [nearly everyday]) they sought news about science via online only news sites (e.g., Slate, HuffPost) (*M* = 2.67, *SD* = 1.44), blogs (*M* = 2.01, *SD* = 1.10), online forums / message boards / wikis (*M* = 2.05, *SD* = 1.18), social networking sites (e.g, Facebook, LinkedIn) (*M =* 2.05, *SD* = 1.34), micro-blogging sites (e.g., Twitter, Tumblr) (*M =* 1.40, *SD* = .92), and video-sharing sites (e.g., YouTube, Vine) (*M =* 1.96, *SD* = .96). A composite measure of online consumption of science news was then constructed (Cronbach’s α = .75, *M* = 2.01, *SD* = .77). Confirmatory Factor Analysis (CFA) with Mplus was used to ensure that the two factor media use measurement was consistent with the data (Chi-square = 82.00 [*df* = 36, *p* < .00], RMSEA = .06 [90%CI: .04-.07], CFI = .94, SRMR = .04).

We controlled for scientists’ past online engagement behavior by asking respondents to indicate about how many total days per year (on a seven-point scale from 1 [0 days] to 7 [>11 days]) they devoted to engagement with adults who are not scientists through websites, blogs, and/or social networks (*M* = 2.59, *SD* = 2.14).

S1 Table provides the descriptive statistics for the endogenous variables used in the multivariate analyses, including reliability for items measured using multiple questions. Scientists’ prioritization of five different communication objectives for online public engagement—inform, excite, build trust, tailor messages, and defend science—represent the criterion variables. S2 Table shows the correlations between communication objectives and S3 Table provides the results of a CFA used to ensure that the five objectives identified from the literature were relatively distinct from each other. Predictor variables include scientific field, attitudes (including personal enjoyment, perceived distributive and procedural unfairness, and perceived ethicality of communication objectives) efficacy (including communication training and external and internal objective-specific efficacy), and norms (including subjective and descriptive norms, and perceptions of colleagues’ prioritization of communication objectives).

## Results

### Descriptive results

**Fig 1:**
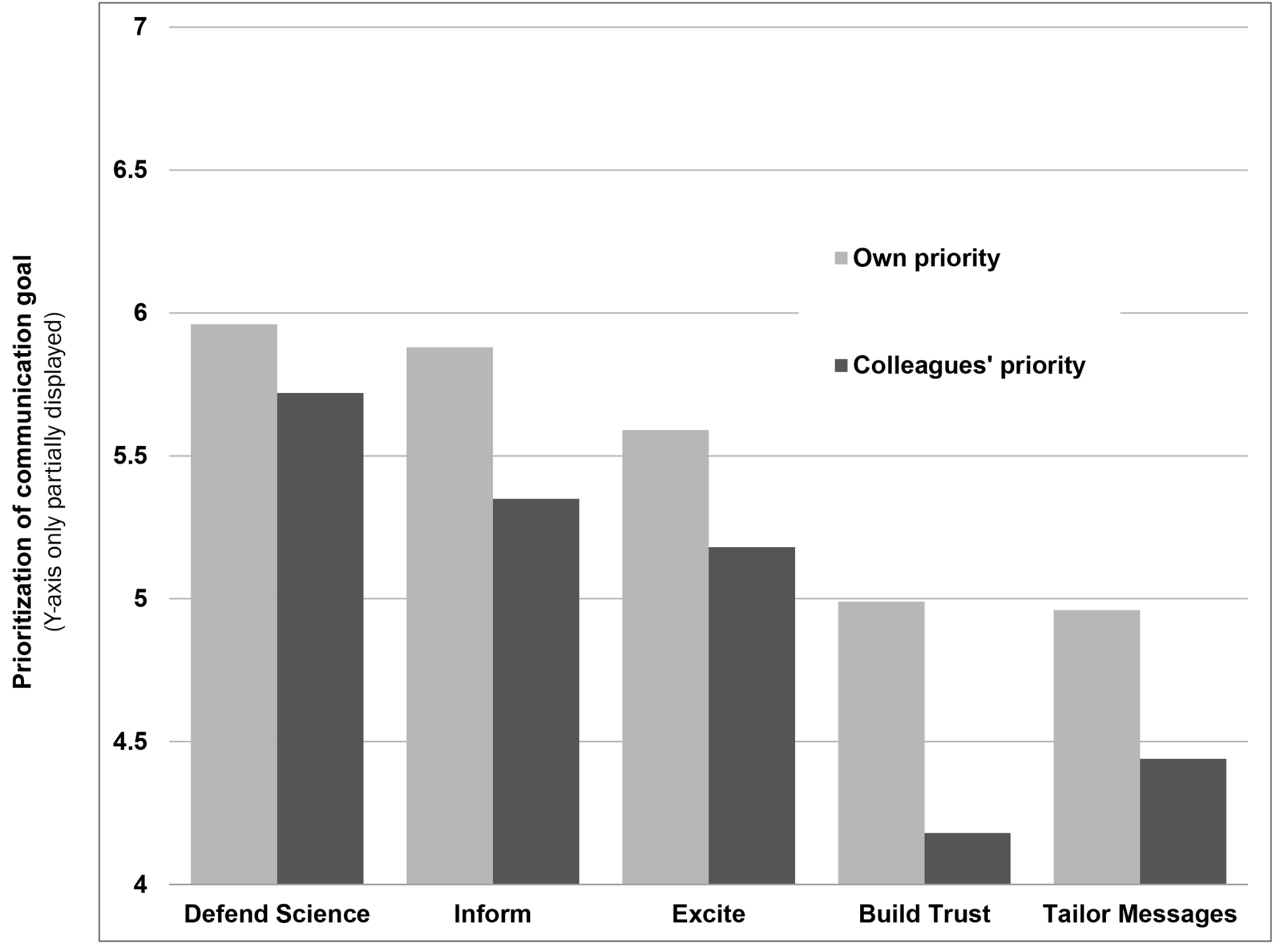
Scientists’ prioritization of five communication objectives for online public engagement (1 = lowest priority, 7 = highest priority).

The first objective of this study was to evaluate scientists’ assessment of five specific objectives for online public communication (S1 Dataset). Fig 1 shows what specific communication objectives scientists prioritize for online public engagement (RQ1), as well as what communication objectives scientists say they think their colleagues prioritize (RQ2). Overall, the mean scores for each objective was above the scale mid-point (4), indicating that scientists personally prioritize each of these objectives and think their colleagues share a relatively similar prioritization. Respondents ranked ‘defending science’ as their own highest priority and also indicated that they think their colleagues also see this objective as the most important. The scientists also prioritized ‘informing the public about science’ highly for themselves and their colleagues. ‘Exciting the public about science’ fell next. ‘Building trust’ and ‘tailoring messages’ were the two least-prioritized communication objectives and but these were prioritized somewhat differently. Respondents indicated that they believe that their colleagues, unlike themselves, prioritize tailoring messages more than building trust.

Closer inspection also reveals that AAAS members believe that they prioritize each of the specific communication objectives more than their scientific colleagues. Paired-sample t-tests (see Table 1) reveal that the surveyed scientists’ are significantly more likely to believe that they prioritize each communication objective more than their colleagues.

**Table 1:** Paired-sample t-tests between scientists’ and colleagues’ prioritizations of communication objectives for online public engagement (1 = lowest priority, 7 = highest priority).

	Personal prioritization		Colleagues’ prioritization				
	Mean	SD	Mean	SD	*df*	*t*	Sig.
Defend	5.96	1.26	5.72	1.35	368	3.85	< .001
Inform	5.88	1.07	5.35	1.33	375	8.27	<
Excite	5.60	1.40	5.18	1.49	376	5.53	< .001
Build trust	5.03	1.27	4.18	1.43	371	12.19	< .001
Tailor messages	4.99	1.38	4.43	1.43	369	7.26	< .001

S1 Table also includes descriptive results that, while not a primary focus of the current analysis, may be of interest. For example, the findings suggest that most scientists do not think that his or her research is likely to be hurt by engaging online (i.e., distributive unfairness). This can be seen in looking at the scale mean for distributive unfairness, which is well below the mid-point of the seven-point (1–7) scale (*M* = 3.15, *SD* = 1.27). On the other hand, many scientists appear that he or she would be treated poorly (i.e., procedural unfairness) when engaging online, as seen in a mean just above the scale mid-point (*M* = 4.37, *SD* = 1.25). Similarly, on average, scientists were somewhat mixed in the degree to which they worried about whether their colleagues would see them in a negative light if they were to engagement (i.e., subjective norm). In this case, the mean was just above the scale mid-point (*M* = 4.25, *SD* = 1.01). See S1 Table for additional details.

### Multivariate results

The primary objective of this study was to identify the factors associated with scientists’ prioritization of specific objectives for online public communication (RQ3). To address this objective, we constructed and tested ordinary least square regression models for each of the five objectives (see Table 2). This approach enabled us to see if different factors drive scientists to prioritize different objectives for online communication. Each regression model explains slightly more than a third of the variance, although the specific variables that emerged as significant predictors varied somewhat.

**Table 2:** Hierarchical regressions: factors associated with scientists’ prioritization of 5 types of online communication objectives for public engagement.

Criterion Variables:	Defend	Inform	Excite	Build	Tailor
	Science			Trust	Messages
**Block 1: Control Variables**					
Age	-.03	-.07	-.12*	-.11	-.05
Gender (male coded high)	.05	-.10*	-.01	-.01	-.01
Ideology (liberal coded high)	.07	.06	.01	-.01	.04
Research Productivity	.04	.01	.11*	-.02	.02
Career level (senior coded high)	.04	.09	-.06	.12	.09
Science news use, online	.01	-.05	-.14***	-.04	.02
Science news use, traditional	.01	.17***	.10**	.08	.02
Past online engagement experience	-.05	-.01	.08	.04	.01
*Incremental R2 (%)*	3.5	10.4***	7.6***	4.6*	3.2
**Block 2: Scientific Field**					
Biomedicine	.01	-.01	.09	.04	.03
Chemistry	.00	-.10*	.00	-.01	.00
Physics/Astronomy	-.08	-.07	.10*	.01	-.02
Social Science	-.08	-.09	-.05	.06	-.01
*Block 2 R2 with Controls (%)*	5.0	13.0*	11.1*	6.0	4.2
**Block 3: Attitudes**					
Fair/Unfair: External procedural	.14**	.12**	-.04	.04	-.05
Fair-Unfair: External distributive	.07	.07	.13**	.05	.13**
Personal Enjoyment	.01	.13***	.02	.09*	.06
Objective ethicality (objective-specific)	.20***	.22***	.19***	-.02	.23***
*Block 3 R2 with Controls and Field (%)*	21.7***	27.7***	20.5***	8.2	21.2***
**Block 4: Efficacy**					
Communication Training	-.03	.03	.06	.08	.01
External efficacy (objective-specific)	.20***	.02	.12**	.15**	.12**
Internal efficacy (objective-specific)	.14**	.05	.16**	.13**	.14**
*Block 4 R2 with Controls and Field (%)*	23.1***	19.2***	22.5***	20.0***	21.0***
**Block 5: Norms**					
Subjective norms	.07	.16***	.00	-.01	-.01
Descriptive norms	.10*	.14***	.06	.08	.07
Perception of colleagues’ communication priorities (objective-specific)	.27***	.29***	.37***	.44***	.28***
*Block 5 R2 with Controls and Field (%)*	26.4***	31.8***	29.5***	30.0***	22.4***
**All Blocks**					
Total R2 (%)	40.0***	40.8***	41.0***	36.9***	35.3***
Total Adjusted R2 (%)	36.4***	37.3***	37.4***	33.1***	31.4***
ANOVA	F^22,389^	F^22,389^	F^22,389^	F^22,389^	F22,389
	11.10***	11.49***	11.56***	9.75***	9.08***

Notes: This table depicts the results of hierarchical ordinary least squares (OLS) regression analysis. The R^2^ for block 2 is after initial controls and the R^2^ for blocks 3–5 include blocks 1–2, but not the other blocks. Total R^2^ includes all five blocks. Each column depicts the final model for each of the five criterion variables, showing which predictor variables are significantly related to each criterion variable while controlling for the effects of all the other predictor variables in the model. The cell entries in each column are standardized regression coefficients.

* *p* < .05

** *p* < .01

*** *p* < .001

#### Control variables

Overall, basic demographics were seldom associated with scientists’ prioritization of specific online public communication objectives. The results suggest that female scientists were slightly more likely to prioritize informative communication and that younger scientists were slightly more likely to prioritize communication designed to excite public audiences. Scientists’ research productivity was associated only with a small increase in prioritization of communication designed to excite people about science. Scientists’ prioritization was not associated with their previous experience participating in online communication, their political ideology, or their career level.

Scientists’ consumption of news about science in online and traditional media platforms was also not associated with how they prioritized online communication designed to build trust, tailor messages, or defend science. However, scientists who consumed more news about science through traditional platforms (e.g., TV, newspapers) were somewhat more likely to prioritize online communication efforts designed to inform and excite public audiences about science. Similarly, scientists who consumed more news about science through online platforms (e.g., blogs, social networks) were less likely to prioritize online communication efforts focused on sowing public excitement about science.

#### Scientific field

Overall, scientists’ disciplines tended to be uncorrelated with views about engagement objectives. There were two minor exceptions: chemists were somewhat less likely than scientists in other fields to say they prioritize public communication designed to inform, and physicists and astronomers were more likely to say they prioritize communication designed to excite public audiences.

#### Attitudes

With regard to general attitudes toward online engagement, scientists who thought they were more likely to be treated unfairly by the public when engaging online (i.e., procedural unfairness) were more likely to prioritize online communication efforts that aim to inform the public about science and defend science from misinformation. In contrast, respondents who thought scientists are likely to have their professional status undermined by engaging in online communication (i.e. distributive unfairness) were more likely to prioritize online communication that focused on exciting the public about science and tailoring messages that connect with their audiences. Neither type of perceived fairness was associated with the communication objective of building public trust regarding science. In addition, scientists who expressed a high level of personal enjoyment for public communication were more likely to prioritize efforts to inform and build public trust about science.

Scientists were also considerably more likely to prioritize specific online communication objectives when they saw these specific objectives as being ethically acceptable. With the exception of trust building, scientists’ ethical acceptance of the other four communication objectives was strongly associated with regarding each objective as important. These associations were some of the strongest relationships revealed by our analyses

#### Efficacy

In most cases, scientists with greater perceived ability (internal efficacy) and positive beliefs relative to the effectiveness of specific online communication objectives (external efficacy) were more likely to prioritize those specific online communication objectives. For example, scientists who felt skilled at building public trust about science and who felt that these efforts would be effective also prioritized efforts to build trust via online public communication. This same relationship existed for the objectives of exiting, tailoring messages, and defending science. The exception to this pattern was that internal and external efficacy were not related to the objective of informing the public. Scientists’ amount of formal communication training was not associated with prioritizing any of the specific objectives.

#### Perceived social norms

Scientists’ broad normative beliefs about public communication were minimally associated with their prioritization of communication objectives. Specifically, scientists who believed that their colleagues engage in (i.e., descriptive norms) and support (i.e., subjective norms) public communication tended to prioritize informative online communication efforts, while scientists who perceived that their colleagues engage were also more likely to prioritize communication efforts to defend science. In contrast, scientists were considerably more likely to prioritize specific online communication objectives when they believed that their colleagues also prioritized these same specific objectives. These relationships were strong and cut across all five communication objectives.

Overall, the multivariate results identify several common factors associated with scientists’ prioritization of specific objectives for online public communication (particularly attitudes about ethics as well as efficacy beliefs) and also suggest that certain factors may matter more for specific objectives. Notably, prioritization of the ‘inform’ objective was predicted by a number of factors that were not associated with the other communication objectives, specifically gender, consumption of science news via traditional media platforms, scientific field, attitude (personal enjoyment), and subjective and descriptive norms. Furthermore, prioritization of the information objective was the only objective not significantly associated with external and internal self-efficacy.

## Discussion

This study aimed to extend research that explores scientists’ as public communicators by assessing the communication objectives that scientists say underlie their engagement activities. Specifically, we sought to assess how one population of scientists prioritized the objective of informing the public, exciting the public, building public trust about science, tailoring messages about science, and defending science from public misinformation. Several limitations of the study are, however, important to highlight before a discussion about potential implications and next steps.

### Limitations

One key limitation is that our results should be considered within the strengths and weaknesses of our sample. On one hand, our sample composition limits the generalizability of our findings. For example, our sample excluded student scientists at a time when understanding student scientists’ relationship with public communication is becoming more relevant given the growth in university-level science communication courses becoming available to them [5,62,63]. It also excluded scientists from industry. However, a primary benefit of sampling the AAAS membership population is that it is one of the world’s largest organization of scientists and one from which there is an active effort to recruit scientists who might want to engage. Although we would advocate that future research find ways to examine increasingly more diverse population of scientists (especially in terms of age), it is unlikely that the underlying relationships explored in our analyses would become irrelevant in other large samples composed of scientists. And, while the risk of non-response bias is legitimate, this phenomenon is a commonplace challenge when studying scientist communicators [6]. This is why we included a modest incentive structure in our survey. Overall, we believe that the strengths of our sample composition outweigh its weaknesses.

It is also important to note that the objectives chosen for this study do not represent an exhaustive list of scientists’ objectives for public communication. The specific selections stemmed from the need to choose a finite number of objectives coupled with consideration of research, theory, and ongoing discussion with scholars and science communication practitioners. We were particularly interested in examining how scientists prioritize more traditional objectives (e.g., informing audiences and defending science) from objectives that are arguably more strategic (e.g., building trust and excitement, framing messages). Additional research is needed to refine concepts and identify additional relevant communication objectives. As noted above, it will also be important to further explore how communication objectives may be linked to the ultimate ends (e.g., support for policy) that science communicators hope to achieve through better communication. Qualitative methods could be particularly useful, especially interviews with scientists and science communication trainers.

Others limitations include the fact that the current study focused on online communication and the nature of the measurement. Additional research should assess whether the results presented would hold in other contexts and using alternative measurement. While the current work used measured adapted from other studies, work should also go into refining measures for use with scientists.

### Implications and future research

It appears that scientists rate defending science as the most pressing communication objective for online public engagement. The surveyed scientists also said they believe that their colleagues most prioritize this objective. While the current data do not specifically indicate that scientists are being excessively aggressive, this situation represents a clear area for future research. Climate scientist Judith Miller, for example, has critiqued aggressive approaches to defending science as an attempt to “circle the wagons” and “point [the] guns outward” by engaging in “ad hominem” attacks on skeptics [64]. And social science research suggests that uncivil messages are likely to be perceived of us as less fair, less informative, and less important [51], as well as generally less credible [53]. Although scientists may be justified in questioning the motives of those who reject scientific consensus (for a discussion, see: [65,66]), even well intentioned attempts to actively “defend” science may have unintended consequences for science-citizen relationships.

Scientists also indicated that they continue to prioritize public communication designed to inform. This is not surprising given that this has traditionally been the chief motivation for scientists to communicate with the public. The risk is that this education focus may come at the expense of other communication objectives. For example, scientists who are focused on ensuring that a public is informed may allot less time to listening to what non-scientists think or producing creative messages designed to capture attention or imagination. Indeed, the results suggest that the scientists surveyed least prioritized the objectives that arguably represent those which may be most likely to lead to positive engagement outcomes: building trust and tailoring messages. This is a problem because, as noted, past communication research has made a clear case showing that attitudes related to trust are correlates of positive views about science (e.g. [25]), that individuals make sense of science through pre-existing mental schema (e.g., [24,67]), and that scientist communicators could be more effective if they were to use messages that resonate with their audiences [44,68].

In terms of past research, our regression analyses also dovetail with extant research on what predicts scientists’ overall engagement. Specifically, we find that scientists’ prioritization of specific communication objectives are associated with key predictors from the TPB, but not demographics or field. Also, many of these factors perform similarly across nearly all of the criterion variables, as might be hoped given the prominence of the variables used in the behavior change literature. Indeed, these similarities point to the value of drawing on the TPB. Differences were, nevertheless, seen for the information objective and it might be useful to try and further determine what about this objective is different. It may be, for example, that this objective is just so ingrained into scientists’ way of thinking about communication that issues of efficacy just do not matter. The finding that the informing objective was the only objective predicted by norms, but not efficacy, may support this argument inasmuch as this suggests scientists’ prioritization may be coming from their social environment, rather than beliefs about capacity or impact.

A number of the attitudinal measures also appear to be meaningful predictors of specific objectives. Specifically, it is noteworthy that AAAS scientists who held more negative views about the fairness of the people that the scientists’ thought they would encounter online was associated with prioritizing informing and defending. This might suggest that those who have more negative views about non-scientists are simply more comfortable engaging in traditional ways that may seem less likely to involve substantial back-and-forth discussion. This interpretation also seems consistent with the finding that ‘defending’ science is the most highly prioritized objective inasmuch as the result suggests that scientists are prioritizing a defensive approach when they perceive their likely online audience as unfair. In contrast, scientists who think that they risk getting hurt through engagement (i.e., perceived distributive unfairness) seem more likely to focus on getting the public excited and tailoring messages to the others’ perceived values. It may be that the concern about being hurt results in a perceived need for a more strategic outlook or an approach that does not depend on gaining acceptance of specific facts.

Also related to attitudes is the strength of the ethicality variable. The results make it clear that perceived ethicality is associated with perceived priority for most objectives. This could further be understood to suggest that any attempt to get scientists to consider increasing the priority of specific objectives must address potential ethical concerns. It may thus be useful to further study the underlying nature and origin of communication-related ethical concerns, although the relatively high means for perceived ethicality suggest that most of the scientists surveyed were relatively comfortable with most objectives about which they were asked. Nevertheless, in some cases, it may be that additional experience with communication might assuage any remaining uneasiness or that, for some scientists, objectives related to building trust or reframing issues will simply never seem ethical. The counter argument might be that things that we need to do to build trust (i.e., treat people with respect, honestly listen to their concerns, behave honestly) are generally ethically acceptable and thus reasonable to encourage. The danger is that someone might attempt to feign respect or listening, rather than find a way to behave in a genuine way. At minimum, it is likely impossible to communicate without conveying trust-relevant content [69] or in a way that frames an issue in one or more ways [44]. Communication training may therefore need to try to help scientists avoid communicating something they do not intend to communicate (e.g., coldness, incompetence, or non-relevant ways of looking at issues). It may also be useful to further explore how different question wordings or different contexts might affect reported ethicality for specific communicaiton objectives.

For norms, it is noteworthy that believing that one’s colleagues are engaging online (descriptive norms) and thinking that one’s colleagues prioritize engaging online (subjective norms) were only associated with seeing information dissemination as a priority. This suggests that general views about engagement are only weakly associated with views about engagement objectives. In contrast, however, the results also indicate that scientists’ own priorities are heavily correlated with what they think their colleagues’ prioritize. This might suggest that any attempt to get scientists to think about different objectives may need to address scientists’ beliefs about their colleagues’ preferred objectives but it could also just mean that scientists think their colleagues are already like them.

For efficacy, the results indicate that scientists are more likely to prioritize objectives that they believe are likely to have an impact and objectives for which they feel they have the skills. These results may be useful because they point to the value of demonstrating the impact of specific objectives and the need to ensure that scientist communicators feel that they can do what it takes to achieve those results. The internal efficacy result could also suggest that scientists may choose objectives based on what they believe they are good at. In any case, addressing both types of efficacy is something that trainers can clearly incorporate into their efforts.

Finally, it is important to reiterate that our data bears on scientists’ online public engagement and not, necessarily, more traditional forms of engagement (e.g., face-to-face, via media, etc.). We focused on this modality of engagement because it as in increasingly central platform for interactions between scientists and the public [70–72] and because our research partner (AAAS) therefore expressed an interest in learning more about how its members were thinking about the subject. Given the conceptual focus of our study on better understanding scientists’ communication objectives (not the particular platform), it will be important to juxtapose the extent to which our findings hold across different types of engagement through additional research. It is noteworthy, in this regard, that many of the predictor variables that are associated with our criterion variables are also significant predictors in other quantitative public engagement research. Although this result is notable, no research has yet examined how scientists’ prioritize communication objectives for more traditional modalities of outreach. Theory and extant research leads us to believe that this research would reveal similar findings to those presented here but it remains a salient empirical question. Research might also seek to examine the objectives or goals that audiences bring to their interactions with scientists, the extent to which their goals complement scientists’ objectives, and how any (di)similarities might affect the communication experience.

## Conclusion

Overall, our findings provide an initial sense of the effects scientists say they are hoping to have when they choose to engage with the public online. One key take home message from this work is that communication researchers and practitioners have an opportunity to help scientists think more reflectively and strategically about the objectives for their public communication efforts. Specifically, as indicated, we hope that our findings can help strengthen efforts to train scientists to become better public communicators. Many of the high-profile organizations trying to improving scientists’ engagement capacity acknowledge that this process involves more than simply teaching scientists’ specific communication skills and tactics. A National Academy of Sciences panel, for example, recently highlighted the challenge of getting scientists to think about communication processes in a systematic way [73]. Similarly, COMPASS, an organization that specializes in building scientists’ communication abilities, includes getting scientists “to know their audience” as a core science communication competency [74]. The current research suggests that, for science communication, ‘knowing your audience’ may mean being thoughtful about what types of impacts we are hoping or expecting to have on those with whom we are communicating and the logic of how we think those impacts are most likely to occur. Our findings thus speak to the potential value of ensuring that communication trainers consider scientists’ communication objectives and ensure that training helps scientists select communication objectives for particular contexts and audiences.

## Supporting Information

S1 Dataset (SAV)

S1 Table Descriptive statistics for endogenous variables used in multivariate analyses. (DOCX)

S2 Table Correlation matrix for the objectives items used to create the criterion variables. Prior to conducting Confirmatory Factor Analysis (CFA), the correlation matrix for the nine items used to measure scientists’ reported communication objectives was produced. It shows that the highest correlations were for the pairs of questions we expected to be most highly correlated.
(DOCX)

S3 Table CFA model comparisons for the objectives items used to create criterion variables. Confirmatory Factor Analysis was conducted via Mplus with the nine items used to measure scientists’ reported communication objectives. Two items were available for four of the objectives (Defend Science, Inform, Build Trust, and Tailor Message) but only one item was available for the ‘Excite’ objective. For the CFA, multiple combinations of the available items were considered. These results suggested that the best model was a four-factor model where the “Excite” question was excluded from the model, indicating that it is better to analyze this measure separately. Overall, these analyses support the underlying research’s focus on five separate communication objectives.
(DOCX)
