# Supplementary material for: Scientists’ Prioritization of Communication Objectives for Public Engagement
Source: PLoS One. 2016 Feb 25;11(2):e0148867. doi: 10.1371/journal.pone.0148867 (PMC4767388; doi:10.1371/journal.pone.0148867)
Supplement: S1 Table — (DOCX) [file pone.0148867.s002.docx]

| **Question topics, wording, measurement, & reliability scores (where appropriate)** | **Mean** | **SD** |
| --- | --- | --- |
| **Communication Objectives**  *“How much should each of the following be a priority for online public engagement with adults who are not scientists?” (7-point scale: 1=lowest priority; 7=highest priority)*  **Defend science (*r* = 0.63, *p*<.001)**  Correcting scientific misinformation.  Defending science from those who spread falsehoods.  **Inform (*r* = 0.41, *p* <.001)**  Ensuring that people are informed about science.  Ensuring the scientists’ findings are part of the public debate.  **Excite**  Getting people excited about science.  **Build trust (*r* = 0.54, *p* <.001)**  Hearing what others think about scientific issues.  Demonstrating the scientific community’s openness and transparency.  **Tailor messages (*r* = 0.54, *p* <.001)**  Framing research implications so they resonate with people’s values.  Describing scientific findings in a ways that make them relevant to specific people.  **Colleagues’ Communication Objectives**  *“Earlier in the survey, we asked about what goals you saw as a priority when it comes to online public engagement. Now, how big a priority to do you think each of the following would be for your colleagues?” (7-point scale: 1=lowest priority; 7=highest priority)*  **Defend science (*r* = 0.75, *p*<.001)**  Correcting scientific misinformation.  Defending science from those who spread falsehoods.  **Inform (*r* = 0.67, *p* <.001)**  Ensuring that people are informed about science.  Ensuring the scientists’ findings are part of the public debate.  **Excite**  Getting people excited about science.  **Build trust (*r* = 0.63, *p* <.001)**  Hearing what others think about scientific issues.  Demonstrating the scientific community’s openness and transparency.  **Tailor messages (*r* = 0.58, *p* <.001)**  Framing research implications so they resonate with people’s values.  Describing scientific findings in a ways that make them relevant to specific people.  **Attitude: Personal enjoyment (*Pearson’s r* = 0.40, *p* <.001)**  *“Please indicate your level of agreement or disagreement with the following statement.”*  *(7-point scale: 1=strongly disagree; 7=strongly agree)*  I try to avoid communicating in public if possible (reverse coded)  I enjoy communicating with the public about my research | **5.96**  6.14  5.79  **5.88**  6.04  5.72  **5.59**  **4.99**  4.76  5.22  **4.96**  4.59  5.34  **5.34**  5.33  5.35  **5.35**  5.52  5.17  **5.18**  **4.18**  3.99  4.36  **4.44**  4.14  4.74  **5.34**  5.33  5.35 | **1.26**  1.22  1.56  **1.07**  1.20  1.35  **1.40**  **1.28**  1.44  1.50  **1.38**  1.71  1.45  **1.35**  1.39  1.50  **1.33**  1.46  1.44  **1.49**  **1.44**  1.62  1.56  **1.43**  1.69  1.49  **1.22**  1.51  1.40 |

| **Question topics, wording, measurement, & reliability scores (where appropriate)(continued)** | **Mean** | **SD** |
| --- | --- | --- |
| **Fairness/Unfairness: External** |  |  |
| *“Thinking generally, if a scientist were to engage with adults online how often*  *would her or she …” (7-point scale: 1=almost never; 7=almost always)*  **Distributive (*alpha* = .93)**  See his/her research hurt | **3.15**  3.10 | **1.27**  1.38 |
| See his/her career hurt | 3.13 | 1.36 |
| See his/her reputation hurt | 3.22 | 1.35 |
| **Procedural (*alpha* = .86)** | **4.37** | **1.25** |
| Be treated rudely | 4.05 | 1.47 |
| Be personally attacked | 4.07 | 1.48 |
| Be misunderstood | 5.02 | 1.25 |
| **Norms: Subjective (*r* = .83, *p* <.001)** | **4.25** | **1.01** |
| *“Thinking generally, if a scientist were to engage with adults online how likely*  *would they be …” (7-point scale: 1=not at all likely; 7=very likely)*  Well regarded by his/her peers. | 4.23 | 1.05 |
| Approved of by his/her colleagues. | 4.28 | 1.05 |
|  |  |  |
| **Norms: Descriptive (*r* = .61, *p* <.001)** | **5.35** | **1.26** |
| *“And overall …” (7-point scale: 1=strongly disagree; 7=strongly agree)*  Most scientists take part in online public engagement | 5.57 | 1.26 |
| My colleagues take part in online public engagement | 5.14 | 1.54 |
|  |  |  |
| **Efficacy: External** |  |  |
| *“How effective do you think online public engagement could actually be in each of*  *the following areas with adults?” (7-point scale: 1=not at all effective; 7=very effective)*  **Defend Science** **(*r* = .79, *p* <.001)**  Correcting scientific misinformation.  Defending science from those who spread falsehoods.  **Inform (*r* = .71, *p* <.001)**  Ensuring that people are informed about scientific issues. | **4.55**  4.66  4.44  **4.81**  4.84 | **1.64**  1.69  1.77  **1.38**  1.55 |
| Ensuring that scientists’ findings are part of the public debate.  **Excite**  Getting people excited about science.  **Build Trust** **(*r* = .52, *p* <.001)**  Hearing what others think about science.  Demonstrating the scientific community’s openness and transparency.  **Tailor Messages** **(*r* = .69, *p* <.001)**  Framing research implications so they resonate with people’s values.  Describing scientific findings in ways that make them relevant to specific people. | 4.77  **4.93**  **4.52**  4.64  4.41  **4.47**  4.46  5.04 | 1.44  **1.52**  **1.36**  1.52  1.61  **1.43**  1.61  1.50 |

| **Question topics, wording, measurement, & reliability scores (where appropriate)(continued)** | **Mean** | **SD** |
| --- | --- | --- |
| **Efficacy: Internal** |  |  |
| *“If you were to take part in online engagement with adults tomorrow and you wanted*  *to pursue the goals below, how effective do you think you could be?”*  *(7-point scale: 1=not at all effective; 7=very effective)* |  |  |
| **Defend Science** **(*r* = .74, *p* <.001)**  Correcting scientific misinformation.  Defending science from those who spread falsehoods.  **Inform (*r* = .80, *p* <.001)**  Ensuring that people are informed about scientific issues.  Ensuring that scientists’ findings are part of the public debate.  **Excite**  Getting people excited about science.  **Build Trust** **(*r* = .56, *p* <.001)**  Hearing what others think about science.  Demonstrating the scientific community’s openness and transparency.  **Tailor Messages** **(*r* = .67, *p* <.001)**  Framing research implications so they resonate with people’s values.  Describing scientific findings in ways that make them relevant to specific people.  **Ethicality**  *“How ethical do you think each of the following goals are when it comes to online*  *engagement by scientists with adults?”*  *(7-point scale: 1=completely unethical; 7=completely ethical)*  **Defend Science** **(*r* = .79, *p* <.001)**  Correcting scientific misinformation.  Defending science from those who spread falsehoods.  **Inform (*r* = .71, *p* <.001)**  Ensuring that people are informed about scientific issues.  Ensuring that scientists’ findings are part of the public debate.  **Excite**  Getting people excited about science.  **Build Trust** **(*r* = .65, *p* <.001)**  Hearing what others think about science.  Demonstrating the scientific community’s openness and transparency.  **Tailor Messages** **(*r* = .69, *p* <.001)**  Framing research implications so they resonate with people’s values.  Describing scientific findings in ways that make them relevant to specific people.  **Communication Training**  *“How much training in communication research have you had?”*  *(7-point scale: 1=no training; 7=A great deal of training)* | **4.68**  4.86  4.49  **4.42**  4.65  4.18  **4.73**  **4.32**  4.40  4.24  **4.38**  4.04  4.71  **6.59**  6.67  6.51  **6.61**  6.67  6.56  **6.49**  **6.46**  6.47  6.44  **5.75**  5.52  5.98  **2.97** | **1.63**  1.65  1.80  **1.41**  1.50  1.53  **1.62**  **1.41**  1.58  1.52  **1.50**  1.64  1.59  **0.86**  0.84  1.02  **0.81**  0.82  0.90  **1.09**  **0.86**  0.96  1.01  **1.41**  1.70  1.37  **1.85** |
| **Scientific Field**  *“What is your primary field or scientific discipline?” (not selected=0; selected=1)*  **Biological or Medical Sciences**  **Chemistry**  **Physics or Astronomy**  **Social Sciences or Policy** | **0.64**  **0.12**  **0.13**  **0.08** | **0.48**  **0.32**  **0.34**  **0.28** |
