# Supplementary material for: Scientists’ Prioritization of Communication Objectives for Public Engagement
Source: PLoS One. 2016 Feb 25;11(2):e0148867. doi: 10.1371/journal.pone.0148867 (PMC4767388; doi:10.1371/journal.pone.0148867)
Supplement: S2 Table — Prior to conducting Confirmatory Factor Analysis (CFA), the correlation matrix for the nine items used to measure scientists’ reported communication objectives was produced. It shows that the highest correlations were for the pairs of questions we expected to be most highly correlated. (DOCX) [file pone.0148867.s003.docx]

S2 Table: *Correlation matrix for the objectives items used to create the criterion variables.*

|  | A1 | A2 | B1 | B2 | C | D1 | D2 | E1 | E2 |
| --- | --- | --- | --- | --- | --- | --- | --- | --- | --- |
| Defend Science: Correcting scientific misinformation (A1) | 1.00 | **.63** | .36 | .30 | .17 | .23 | .35 | .30 | .34 |
| Defend Science: Defending science from those who spread falsehoods (A2) |  | 1.00 | .18 | .23 | .27 | .16 | .31 | .23 | .22 |
| Inform: Ensuring that people are informed about scientific issues (B1) |  |  | 1.00 | **.41** | .26 | .29 | .27 | .19 | .26 |
| Inform: Ensuring that scientists' findings are part of the public debate (B2) |  |  |  | 1.00 | .23 | .36 | .32 | .25 | .26 |
| Excite: Getting people excited about science (C) |  |  |  |  | 1.00 | .38 | .32 | .25 | .33 |
| Build Trust: Hearing what others think about scientific issues (D1) |  |  |  |  |  | 1.00 | **.54** | .37 | .35 |
| Build Trust: Demonstrating the scientific community's openness and transparency (D2) |  |  |  |  |  |  | 1.00 | .34 | .28 |
| Tailor: Framing research implications so they resonate with people's values (E1) |  |  |  |  |  |  |  | 1.00 | **.53** |
| Tailor: Describing scientific findings in ways that make them relevant to specific people (E2) |  |  |  |  |  |  |  |  | 1.00 |

Notes: All correlations *p* > .00; N = 379-385.
