# Supplementary material for: Scientists’ Prioritization of Communication Objectives for Public Engagement
Source: PLoS One. 2016 Feb 25;11(2):e0148867. doi: 10.1371/journal.pone.0148867 (PMC4767388; doi:10.1371/journal.pone.0148867)
Supplement: S3 Table — Confirmatory Factor Analysis was conducted via Mplus with the nine items used to measure scientists’ reported communication objectives. Two items were available for four of the objectives (Defend Science, Inform, Build Trust, and Tailor Message) but only one item was available for the ‘Excite’ objective. For the CFA, multiple combinations of the available items were considered. These results suggested that the best model was a four-factor model where the “Excite” question was excluded from the model, indicating that it is better to analyze this measure separately. Overall, these analyses support the underlying research’s focus on five separate communication objectives. (DOCX) [file pone.0148867.s004.docx]

S3 Table: CFA model comparisons for the objectives items used to create criterion variables.

| Model | Chi Square | RMSEA [90%CI] | CFI | SRMR | AIC |
| --- | --- | --- | --- | --- | --- |
| Two factors (A1-B2, C-E2) | 296.40 (*df* = 26, *p* < .00) | .16 [.14-.17] | .72 | .08 | 12866.54 |
| One factor | 297.97 (df = 27, *p* < .00) | .15[.14-.17] | .72 | .08 | 12866.11 |
| Four factors (excite with ‘tailor…’) | 86.39 (*df* =21, *p* < .00) | .09 [.07-.10] | .93 | .04 | 12666.53 |
| Four factors (excite with ‘… trust’) | 67.20 (*df* = 21, *p* <.00) | .07 [.05-.09] | .95 | .04 | 12647.34 |
| Four factors (no excite) | 34.09 (*df* = 14, *p* < .00) | .06 [.03-.08] | .98 | .08 | 11237.98 |

Notes: N = 379-385.
